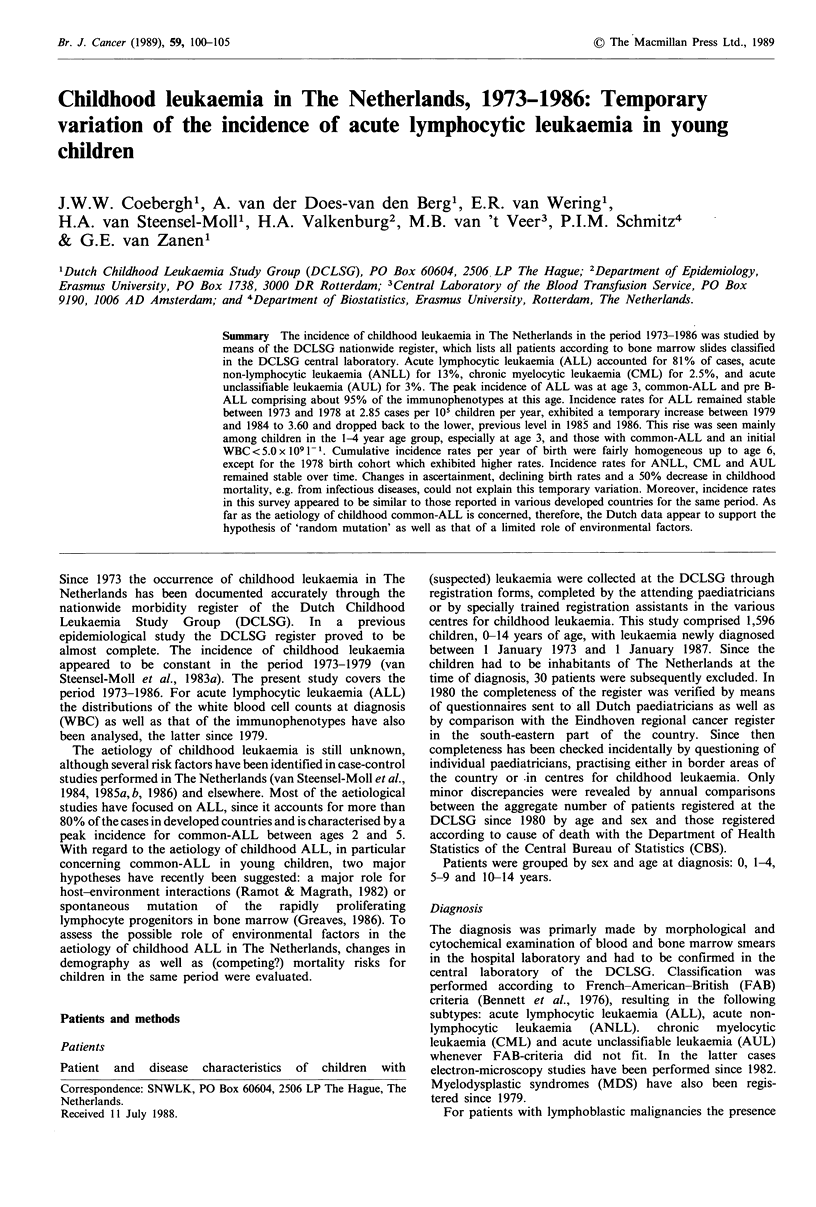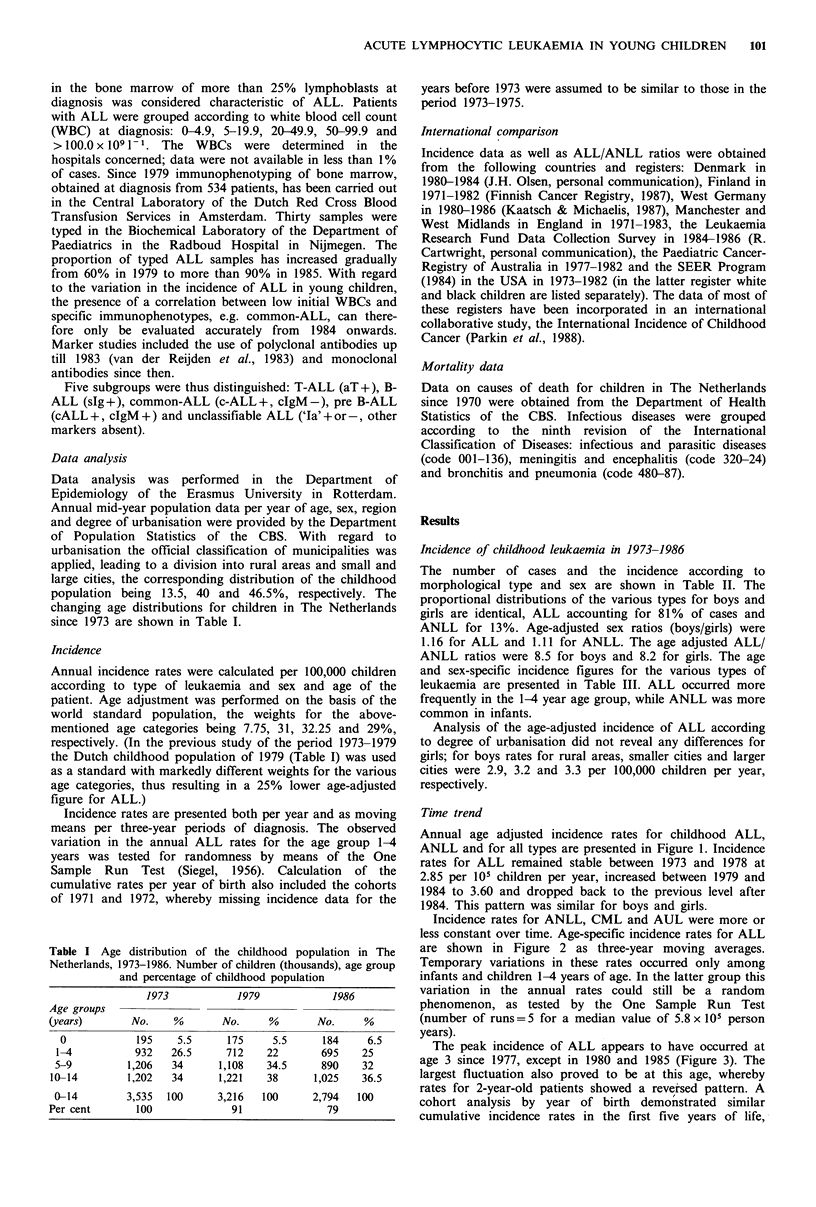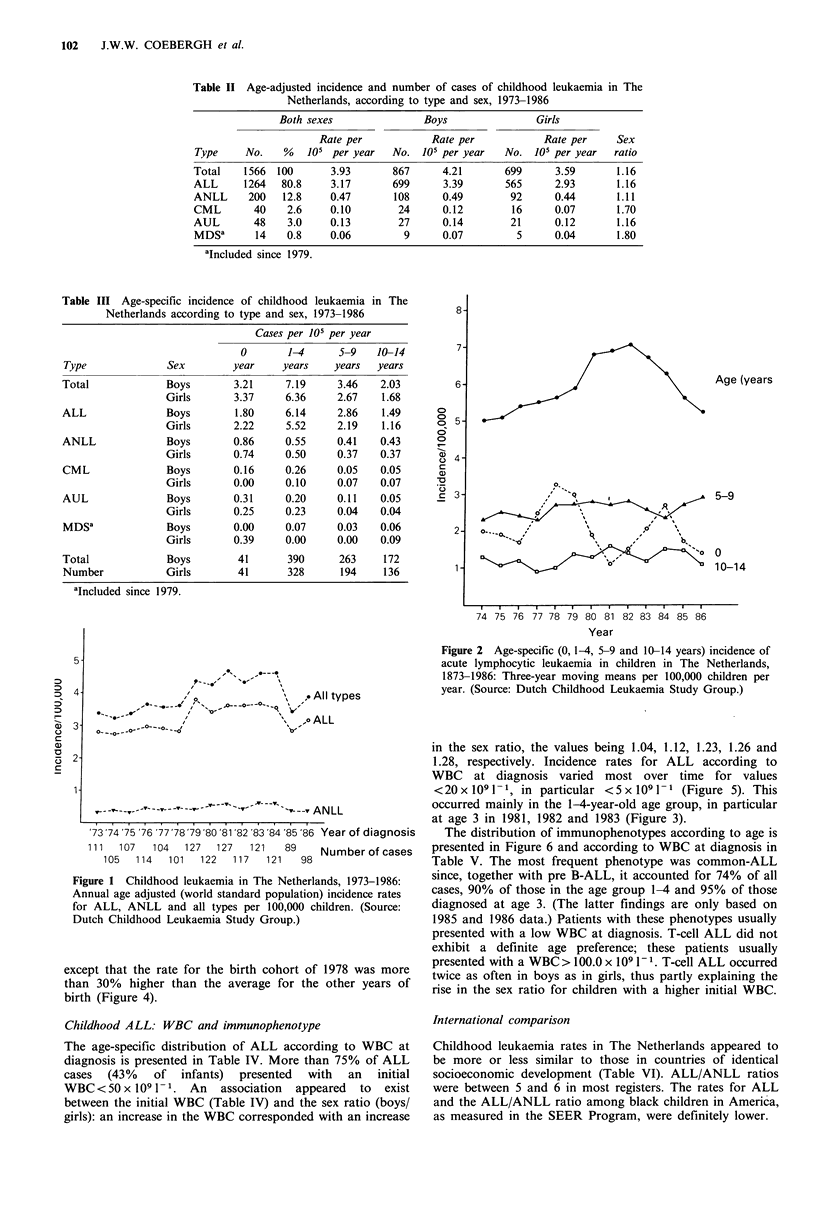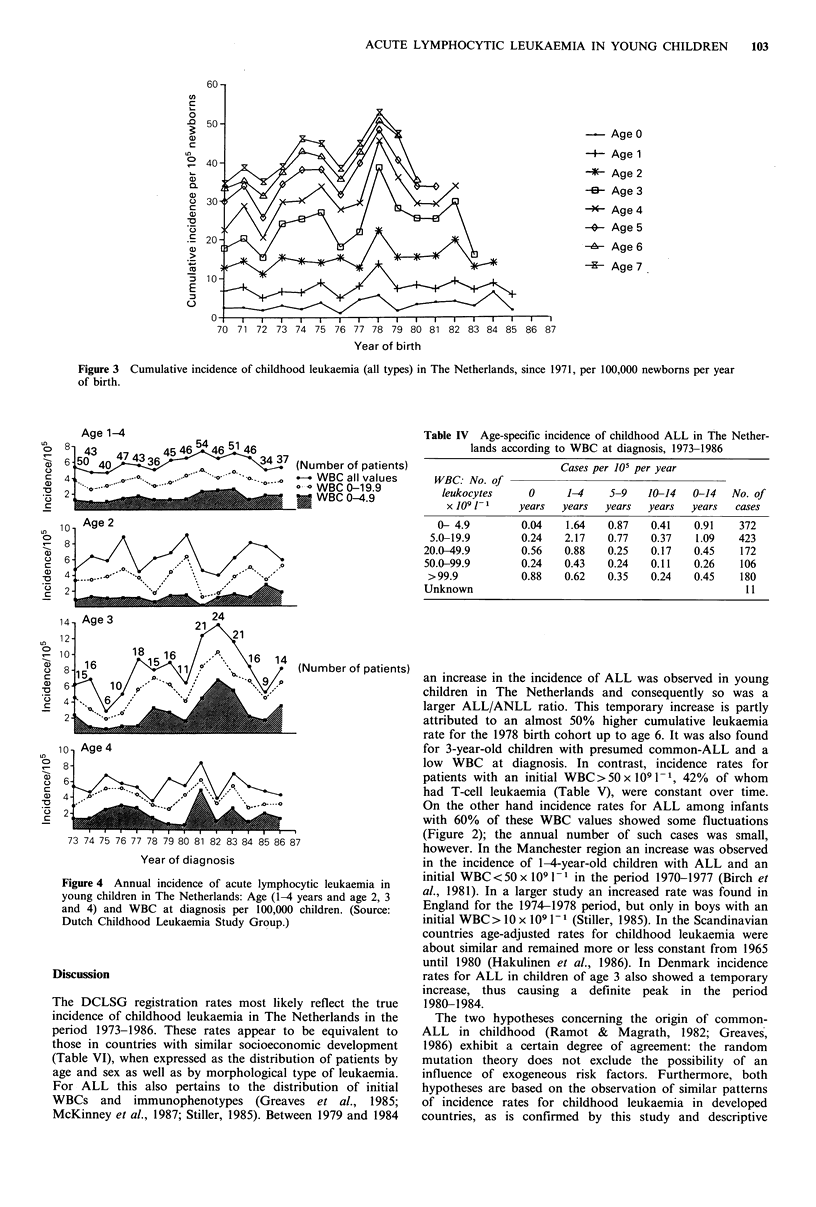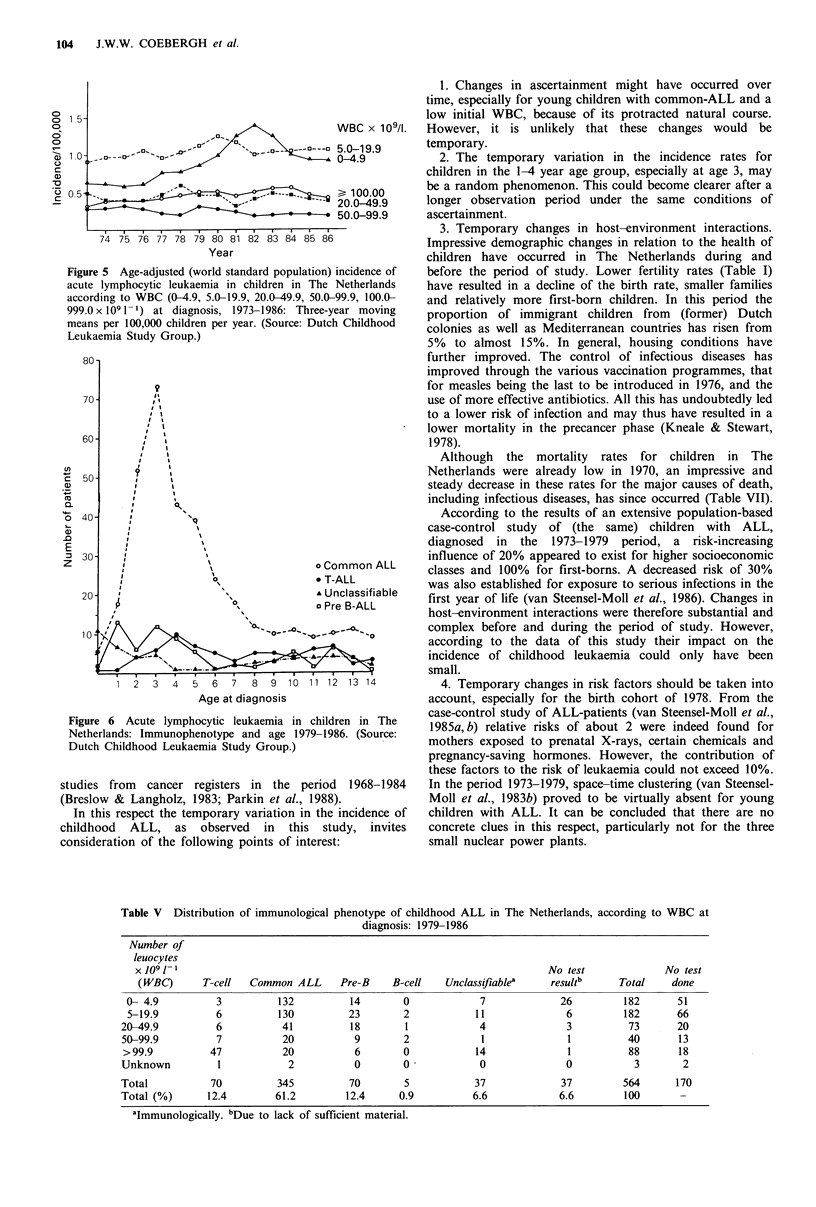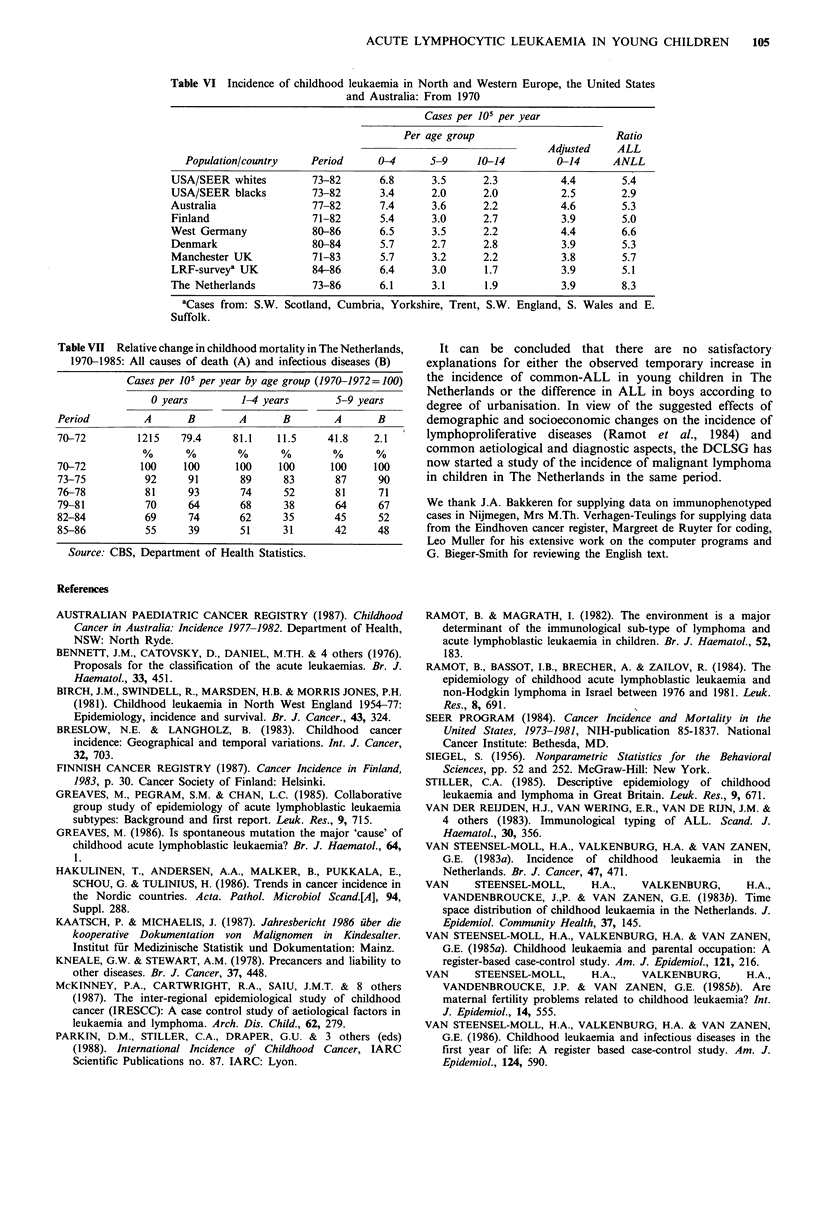# Childhood leukaemia in The Netherlands, 1973-1986: temporary variation of the incidence of acute lymphocytic leukaemia in young children.

**DOI:** 10.1038/bjc.1989.20

**Published:** 1989-01

**Authors:** J. W. Coebergh, A. van der Does-van den Berg, E. R. van Wering, H. A. van Steensel-Moll, H. A. Valkenburg, M. B. van't Veer, P. I. Schmitz, G. E. van Zanen

**Affiliations:** Dutch Childhood Leukaemia Study Group, The Hague, The Netherlands.

## Abstract

The incidence of childhood leukaemia in The Netherlands in the period 1973-1986 was studied by means of the DCLSG nationwide register, which lists all patients according to bone marrow slides classified in the DCLSG central laboratory. Acute lymphocytic leukaemia (ALL) accounted for 81% of cases, acute non-lymphocytic leukaemia (ANLL) for 13%, chronic myelocytic leukaemia (CML) for 2.5%, and acute unclassifiable leukaemia (AUL) for 3%. The peak incidence of ALL was at age 3, common-ALL and pre B-ALL comprising about 95% of the immunophenotypes at this age. Incidence rates for ALL remained stable between 1973 and 1978 at 2.85 cases per 10(5) children per year, exhibited a temporary increase between 1979 and 1984 to 3.60 and dropped back to the lower, previous level in 1985 and 1986. This rise was seen mainly among children in the 1-4 year age group, especially at age 3, and those with common-ALL and an initial WBC less than 5.0 x 10(9) l-1. Cumulative incidence rates per year of birth were fairly homogeneous up to age 6, except for the 1978 birth cohort which exhibited higher rates. Incidence rates for ANLL, CML and AUL remained stable over time. Changes in ascertainment, declining birth rates and a 50% decrease in childhood mortality, e.g. from infectious diseases, could not explain this temporary variation. Moreover, incidence rates in this survey appeared to be similar to those reported in various developed countries for the same period. As far as the aetiology of childhood common-ALL is concerned, therefore, the Dutch data appear to support the hypothesis of 'random mutation' as well as that of a limited role of environmental factors.


*Br. J. Cancer* (1989), **59**, 100–105 © The Macmillan Press Ltd., 1989

### Childhood leukaemia in The Netherlands, 1973–1986: Temporary variation of the incidence of acute lymphocytic leukaemia in young children

J.W.W. Coebergh[1], A. van der Does-van den Berg[1], E.R. van Wering[1], H.A. van Steensel-Moll[1], H.A. Valkenburg[2], M.B. van 't Veer[3], P.I.M. Schmitz[4] & G.E. van Zanen[1]

[1]*Dutch Childhood Leukaemia Study Group (DCLSG), PO Box 60604, 2506 LP The Hague;* [2]*Department of Epidemiology, Erasmus University, PO Box 1738, 3000 DR Rotterdam;* [3]*Central Laboratory of the Blood Transfusion Service, PO Box 9190, 1006 AD Amsterdam; and* [4]*Department of Biostatistics, Erasmus University, Rotterdam, The Netherlands.*

**Summary** The incidence of childhood leukaemia in The Netherlands in the period 1973–1986 was studied by means of the DCLSG nationwide register, which lists all patients according to bone marrow slides classified in the DCLSG central laboratory. Acute lymphocytic leukaemia (ALL) accounted for 81% of cases, acute non-lymphocytic leukaemia (ANLL) for 13%, chronic myelocytic leukaemia (CML) for 2.5%, and acute unclassifiable leukaemia (AUL) for 3%. The peak incidence of ALL was at age 3, common-ALL and pre B-ALL comprising about 95% of the immunophenotypes at this age. Incidence rates for ALL remained stable between 1973 and 1978 at 2.85 cases per $10^5$ children per year, exhibited a temporary increase between 1979 and 1984 to 3.60 and dropped back to the lower, previous level in 1985 and 1986. This rise was seen mainly among children in the 1–4 year age group, especially at age 3, and those with common-ALL and an initial WBC $< 5.0 \times 10^9 l^{-1}$. Cumulative incidence rates per year of birth were fairly homogeneous up to age 6, except for the 1978 birth cohort which exhibited higher rates. Incidence rates for ANLL, CML and AUL remained stable over time. Changes in ascertainment, declining birth rates and a 50% decrease in childhood mortality, e.g. from infectious diseases, could not explain this temporary variation. Moreover, incidence rates in this survey appeared to be similar to those reported in various developed countries for the same period. As far as the aetiology of childhood common-ALL is concerned, therefore, the Dutch data appear to support the hypothesis of 'random mutation' as well as that of a limited role of environmental factors.

Since 1973 the occurrence of childhood leukaemia in The Netherlands has been documented accurately through the nationwide morbidity register of the Dutch Childhood Leukaemia Study Group (DCLSG). In a previous epidemiological study the DCLSG register proved to be almost complete. The incidence of childhood leukaemia appeared to be constant in the period 1973–1979 (van Steensel-Moll *et al.*, 1983*a*). The present study covers the period 1973–1986. For acute lymphocytic leukaemia (ALL) the distributions of the white blood cell counts at diagnosis (WBC) as well as that of the immunophenotypes have also been analysed, the latter since 1979.

The aetiology of childhood leukaemia is still unknown, although several risk factors have been identified in case-control studies performed in The Netherlands (van Steensel-Moll *et al.*, 1984, 1985*a*, *b*, 1986) and elsewhere. Most of the aetiological studies have focused on ALL, since it accounts for more than 80% of the cases in developed countries and is characterised by a peak incidence for common-ALL between ages 2 and 5. With regard to the aetiology of childhood ALL, in particular concerning common-ALL in young children, two major hypotheses have recently been suggested: a major role for host–environment interactions (Ramot & Magrath, 1982) or spontaneous mutation of the rapidly proliferating lymphocyte progenitors in bone marrow (Greaves, 1986). To assess the possible role of environmental factors in the aetiology of childhood ALL in The Netherlands, changes in demography as well as (competing?) mortality risks for children in the same period were evaluated.

### Patients and methods

### *Patients*

Patient and disease characteristics of children with (suspected) leukaemia were collected at the DCLSG through registration forms, completed by the attending paediatricians or by specially trained registration assistants in the various centres for childhood leukaemia. This study comprised 1,596 children, 0–14 years of age, with leukaemia newly diagnosed between 1 January 1973 and 1 January 1987. Since the children had to be inhabitants of The Netherlands at the time of diagnosis, 30 patients were subsequently excluded. In 1980 the completeness of the register was verified by means of questionnaires sent to all Dutch paediatricians as well as by comparison with the Eindhoven regional cancer register in the south-eastern part of the country. Since then completeness has been checked incidentally by questioning of individual paediatricians, practising either in border areas of the country or in centres for childhood leukaemia. Only minor discrepancies were revealed by annual comparisons between the aggregate number of patients registered at the DCLSG since 1980 by age and sex and those registered according to cause of death with the Department of Health Statistics of the Central Bureau of Statistics (CBS).

Patients were grouped by sex and age at diagnosis: 0, 1–4, 5–9 and 10–14 years.

### *Diagnosis*

The diagnosis was primarily made by morphological and cytochemical examination of blood and bone marrow smears in the hospital laboratory and had to be confirmed in the central laboratory of the DCLSG. Classification was performed according to French–American–British (FAB) criteria (Bennett *et al.*, 1976), resulting in the following subtypes: acute lymphocytic leukaemia (ALL), acute non-lymphocytic leukaemia (ANLL). chronic myelocytic leukaemia (CML) and acute unclassifiable leukaemia (AUL) whenever FAB-criteria did not fit. In the latter cases electron-microscopy studies have been performed since 1982. Myelodysplastic syndromes (MDS) have also been registered since 1979.

For patients with lymphoblastic malignancies the presence

Correspondence: SNWLK, PO Box 60604, 2506 LP The Hague, The Netherlands.
Received 11 July 1988.

in the bone marrow of more than 25% lymphoblasts at diagnosis was considered characteristic of ALL. Patients with ALL were grouped according to white blood cell count (WBC) at diagnosis: 0–4.9, 5–19.9, 20–49.9, 50–99.9 and $>100.0\times10^{9}\,l^{-1}$. The WBCs were determined in the hospitals concerned; data were not available in less than 1% of cases. Since 1979 immunophenotyping of bone marrow, obtained at diagnosis from 534 patients, has been carried out in the Central Laboratory of the Dutch Red Cross Blood Transfusion Services in Amsterdam. Thirty samples were typed in the Biochemical Laboratory of the Department of Paediatrics in the Radboud Hospital in Nijmegen. The proportion of typed ALL samples has increased gradually from 60% in 1979 to more than 90% in 1985. With regard to the variation in the incidence of ALL in young children, the presence of a correlation between low initial WBCs and specific immunophenotypes, e.g. common-ALL, can therefore only be evaluated accurately from 1984 onwards. Marker studies included the use of polyclonal antibodies up till 1983 (van der Reijden *et al.*, 1983) and monoclonal antibodies since then.

Five subgroups were thus distinguished: T-ALL (aT+), B-ALL (sIg+), common-ALL (c-ALL+, cIgM−), pre B-ALL (cALL+, cIgM+) and unclassifiable ALL ('Ia'+or−, other markers absent).

*Data analysis*

Data analysis was performed in the Department of Epidemiology of the Erasmus University in Rotterdam. Annual mid-year population data per year of age, sex, region and degree of urbanisation were provided by the Department of Population Statistics of the CBS. With regard to urbanisation the official classification of municipalities was applied, leading to a division into rural areas and small and large cities, the corresponding distribution of the childhood population being 13.5, 40 and 46.5%, respectively. The changing age distributions for children in The Netherlands since 1973 are shown in Table I.

*Incidence*

Annual incidence rates were calculated per 100,000 children according to type of leukaemia and sex and age of the patient. Age adjustment was performed on the basis of the world standard population, the weights for the above-mentioned age categories being 7.75, 31, 32.25 and 29%, respectively. (In the previous study of the period 1973–1979 the Dutch childhood population of 1979 (Table I) was used as a standard with markedly different weights for the various age categories, thus resulting in a 25% lower age-adjusted figure for ALL.)

Incidence rates are presented both per year and as moving means per three-year periods of diagnosis. The observed variation in the annual ALL rates for the age group 1–4 years was tested for randomness by means of the One Sample Run Test (Siegel, 1956). Calculation of the cumulative rates per year of birth also included the cohorts of 1971 and 1972, whereby missing incidence data for the years before 1973 were assumed to be similar to those in the period 1973–1975.

**Table I** Age distribution of the childhood population in The Netherlands, 1973–1986. Number of children (thousands), age group and percentage of childhood population

| Age groups (years) | 1973 No. | 1973 % | 1979 No. | 1979 % | 1986 No. | 1986 % |
|---|---|---|---|---|---|---|
| 0 | 195 | 5.5 | 175 | 5.5 | 184 | 6.5 |
| 1–4 | 932 | 26.5 | 712 | 22 | 695 | 25 |
| 5–9 | 1,206 | 34 | 1,108 | 34.5 | 890 | 32 |
| 10–14 | 1,202 | 34 | 1,221 | 38 | 1,025 | 36.5 |
| 0–14 | 3,535 | 100 | 3,216 | 100 | 2,794 | 100 |
| Per cent | 100 | | 91 | | 79 | |

*International comparison*

Incidence data as well as ALL/ANLL ratios were obtained from the following countries and registers: Denmark in 1980–1984 (J.H. Olsen, personal communication), Finland in 1971–1982 (Finnish Cancer Registry, 1987), West Germany in 1980–1986 (Kaatsch & Michaelis, 1987), Manchester and West Midlands in England in 1971–1983, the Leukaemia Research Fund Data Collection Survey in 1984–1986 (R. Cartwright, personal communication), the Paediatric Cancer-Registry of Australia in 1977–1982 and the SEER Program (1984) in the USA in 1973–1982 (in the latter register white and black children are listed separately). The data of most of these registers have been incorporated in an international collaborative study, the International Incidence of Childhood Cancer (Parkin *et al.*, 1988).

*Mortality data*

Data on causes of death for children in The Netherlands since 1970 were obtained from the Department of Health Statistics of the CBS. Infectious diseases were grouped according to the ninth revision of the International Classification of Diseases: infectious and parasitic diseases (code 001–136), meningitis and encephalitis (code 320–24) and bronchitis and pneumonia (code 480–87).

### Results

*Incidence of childhood leukaemia in 1973–1986*

The number of cases and the incidence according to morphological type and sex are shown in Table II. The proportional distributions of the various types for boys and girls are identical, ALL accounting for 81% of cases and ANLL for 13%. Age-adjusted sex ratios (boys/girls) were 1.16 for ALL and 1.11 for ANLL. The age adjusted ALL/ANLL ratios were 8.5 for boys and 8.2 for girls. The age and sex-specific incidence figures for the various types of leukaemia are presented in Table III. ALL occurred more frequently in the 1–4 year age group, while ANLL was more common in infants.

Analysis of the age-adjusted incidence of ALL according to degree of urbanisation did not reveal any differences for girls; for boys rates for rural areas, smaller cities and larger cities were 2.9, 3.2 and 3.3 per 100,000 children per year, respectively.

*Time trend*

Annual age adjusted incidence rates for childhood ALL, ANLL and for all types are presented in Figure 1. Incidence rates for ALL remained stable between 1973 and 1978 at 2.85 per $10^5$ children per year, increased between 1979 and 1984 to 3.60 and dropped back to the previous level after 1984. This pattern was similar for boys and girls.

Incidence rates for ANLL, CML and AUL were more or less constant over time. Age-specific incidence rates for ALL are shown in Figure 2 as three-year moving averages. Temporary variations in these rates occurred only among infants and children 1–4 years of age. In the latter group this variation in the annual rates could still be a random phenomenon, as tested by the One Sample Run Test (number of runs = 5 for a median value of $5.8\times10^5$ person years).

The peak incidence of ALL appears to have occurred at age 3 since 1977, except in 1980 and 1985 (Figure 3). The largest fluctuation also proved to be at this age, whereby rates for 2-year-old patients showed a reversed pattern. A cohort analysis by year of birth demonstrated similar cumulative incidence rates in the first five years of life,

**Table II** Age-adjusted incidence and number of cases of childhood leukaemia in The Netherlands, according to type and sex, 1973–1986

| | *Both sexes* | | | *Boys* | | *Girls* | | |
|---|---|---|---|---|---|---|---|---|
| *Type* | *No.* | *%* | *Rate per $10^5$ per year* | *No.* | *Rate per $10^5$ per year* | *No.* | *Rate per $10^5$ per year* | *Sex ratio* |
| Total | 1566 | 100 | 3.93 | 867 | 4.21 | 699 | 3.59 | 1.16 |
| ALL | 1264 | 80.8 | 3.17 | 699 | 3.39 | 565 | 2.93 | 1.16 |
| ANLL | 200 | 12.8 | 0.47 | 108 | 0.49 | 92 | 0.44 | 1.11 |
| CML | 40 | 2.6 | 0.10 | 24 | 0.12 | 16 | 0.07 | 1.70 |
| AUL | 48 | 3.0 | 0.13 | 27 | 0.14 | 21 | 0.12 | 1.16 |
| MDS[a] | 14 | 0.8 | 0.06 | 9 | 0.07 | 5 | 0.04 | 1.80 |

[a]Included since 1979.

**Table III** Age-specific incidence of childhood leukaemia in The Netherlands according to type and sex, 1973–1986

| | | *Cases per $10^5$ per year* | | | |
|---|---|---|---|---|---|
| *Type* | *Sex* | *0 year* | *1–4 years* | *5–9 years* | *10–14 years* |
| Total | Boys | 3.21 | 7.19 | 3.46 | 2.03 |
| | Girls | 3.37 | 6.36 | 2.67 | 1.68 |
| ALL | Boys | 1.80 | 6.14 | 2.86 | 1.49 |
| | Girls | 2.22 | 5.52 | 2.19 | 1.16 |
| ANLL | Boys | 0.86 | 0.55 | 0.41 | 0.43 |
| | Girls | 0.74 | 0.50 | 0.37 | 0.37 |
| CML | Boys | 0.16 | 0.26 | 0.05 | 0.05 |
| | Girls | 0.00 | 0.10 | 0.07 | 0.07 |
| AUL | Boys | 0.31 | 0.20 | 0.11 | 0.05 |
| | Girls | 0.25 | 0.23 | 0.04 | 0.04 |
| MDS[a] | Boys | 0.00 | 0.07 | 0.03 | 0.06 |
| | Girls | 0.39 | 0.00 | 0.00 | 0.09 |
| Total Number | Boys | 41 | 390 | 263 | 172 |
| | Girls | 41 | 328 | 194 | 136 |

[a]Included since 1979.

**Figure 1** Childhood leukaemia in The Netherlands, 1973–1986: Annual age adjusted (world standard population) incidence rates for ALL, ANLL and all types per 100,000 children. (Source: Dutch Childhood Leukaemia Study Group.)

except that the rate for the birth cohort of 1978 was more than 30% higher than the average for the other years of birth (Figure 4).

### *Childhood ALL: WBC and immunophenotype*

The age-specific distribution of ALL according to WBC at diagnosis is presented in Table IV. More than 75% of ALL cases (43% of infants) presented with an initial WBC < 50 × $10^9$ $l^{-1}$. An association appeared to exist between the initial WBC (Table IV) and the sex ratio (boys/girls): an increase in the WBC corresponded with an increase

**Figure 2** Age-specific (0, 1–4, 5–9 and 10–14 years) incidence of acute lymphocytic leukaemia in children in The Netherlands, 1873–1986: Three-year moving means per 100,000 children per year. (Source: Dutch Childhood Leukaemia Study Group.)

in the sex ratio, the values being 1.04, 1.12, 1.23, 1.26 and 1.28, respectively. Incidence rates for ALL according to WBC at diagnosis varied most over time for values < 20 × $10^9$ $l^{-1}$, in particular < 5 × $10^9$ $l^{-1}$ (Figure 5). This occurred mainly in the 1–4-year-old age group, in particular at age 3 in 1981, 1982 and 1983 (Figure 3).

The distribution of immunophenotypes according to age is presented in Figure 6 and according to WBC at diagnosis in Table V. The most frequent phenotype was common-ALL since, together with pre B-ALL, it accounted for 74% of all cases, 90% of those in the age group 1–4 and 95% of those diagnosed at age 3. (The latter findings are only based on 1985 and 1986 data.) Patients with these phenotypes usually presented with a low WBC at diagnosis. T-cell ALL did not exhibit a definite age preference; these patients usually presented with a WBC > 100.0 × $10^9$ $l^{-1}$. T-cell ALL occurred twice as often in boys as in girls, thus partly explaining the rise in the sex ratio for children with a higher initial WBC.

### *International comparison*

Childhood leukaemia rates in The Netherlands appeared to be more or less similar to those in countries of identical socioeconomic development (Table VI). ALL/ANLL ratios were between 5 and 6 in most registers. The rates for ALL and the ALL/ANLL ratio among black children in America, as measured in the SEER Program, were definitely lower.

**Figure 3** Cumulative incidence of childhood leukaemia (all types) in The Netherlands, since 1971, per 100,000 newborns per year of birth.

**Figure 4** Annual incidence of acute lymphocytic leukaemia in young children in The Netherlands: Age (1–4 years and age 2, 3 and 4) and WBC at diagnosis per 100,000 children. (Source: Dutch Childhood Leukaemia Study Group.)

### Discussion

The DCLSG registration rates most likely reflect the true incidence of childhood leukaemia in The Netherlands in the period 1973–1986. These rates appear to be equivalent to those in countries with similar socioeconomic development (Table VI), when expressed as the distribution of patients by age and sex as well as by morphological type of leukaemia. For ALL this also pertains to the distribution of initial WBCs and immunophenotypes (Greaves *et al.*, 1985; McKinney *et al.*, 1987; Stiller, 1985). Between 1979 and 1984

**Table IV** Age-specific incidence of childhood ALL in The Netherlands according to WBC at diagnosis, 1973–1986

| *WBC: No. of leukocytes* $\times 10^9\,l^{-1}$ | Cases per $10^5$ per year | | | | | |
|---|---|---|---|---|---|---|
| | *0 years* | *1–4 years* | *5–9 years* | *10–14 years* | *0–14 years* | *No. of cases* |
| 0– 4.9 | 0.04 | 1.64 | 0.87 | 0.41 | 0.91 | 372 |
| 5.0–19.9 | 0.24 | 2.17 | 0.77 | 0.37 | 1.09 | 423 |
| 20.0–49.9 | 0.56 | 0.88 | 0.25 | 0.17 | 0.45 | 172 |
| 50.0–99.9 | 0.24 | 0.43 | 0.24 | 0.11 | 0.26 | 106 |
| >99.9 | 0.88 | 0.62 | 0.35 | 0.24 | 0.45 | 180 |
| Unknown | | | | | | 11 |

an increase in the incidence of ALL was observed in young children in The Netherlands and consequently so was a larger ALL/ANLL ratio. This temporary increase is partly attributed to an almost 50% higher cumulative leukaemia rate for the 1978 birth cohort up to age 6. It was also found for 3-year-old children with presumed common-ALL and a low WBC at diagnosis. In contrast, incidence rates for patients with an initial WBC > $50 \times 10^9\,l^{-1}$, 42% of whom had T-cell leukaemia (Table V), were constant over time. On the other hand incidence rates for ALL among infants with 60% of these WBC values showed some fluctuations (Figure 2); the annual number of such cases was small, however. In the Manchester region an increase was observed in the incidence of 1–4-year-old children with ALL and an initial WBC < $50 \times 10^9\,l^{-1}$ in the period 1970–1977 (Birch *et al.*, 1981). In a larger study an increased rate was found in England for the 1974–1978 period, but only in boys with an initial WBC > $10 \times 10^9\,l^{-1}$ (Stiller, 1985). In the Scandinavian countries age-adjusted rates for childhood leukaemia were about similar and remained more or less constant from 1965 until 1980 (Hakulinen *et al.*, 1986). In Denmark incidence rates for ALL in children of age 3 also showed a temporary increase, thus causing a definite peak in the period 1980–1984.

The two hypotheses concerning the origin of common-ALL in childhood (Ramot & Magrath, 1982; Greaves, 1986) exhibit a certain degree of agreement: the random mutation theory does not exclude the possibility of an influence of exogeneous risk factors. Furthermore, both hypotheses are based on the observation of similar patterns of incidence rates for childhood leukaemia in developed countries, as is confirmed by this study and descriptive

**Figure 5** Age-adjusted (world standard population) incidence of acute lymphocytic leukaemia in children in The Netherlands according to WBC (0–4.9, 5.0–19.9, 20.0–49.9, 50.0–99.9, 100.0–999.0 × $10^9$ $l^{-1}$) at diagnosis, 1973–1986: Three-year moving means per 100,000 children per year. (Source: Dutch Childhood Leukaemia Study Group.)

**Figure 6** Acute lymphocytic leukaemia in children in The Netherlands: Immunophenotype and age 1979–1986. (Source: Dutch Childhood Leukaemia Study Group.)

studies from cancer registers in the period 1968–1984 (Breslow & Langholz, 1983; Parkin *et al.*, 1988).

In this respect the temporary variation in the incidence of childhood ALL, as observed in this study, invites consideration of the following points of interest:

1. Changes in ascertainment might have occurred over time, especially for young children with common-ALL and a low initial WBC, because of its protracted natural course. However, it is unlikely that these changes would be temporary.

2. The temporary variation in the incidence rates for children in the 1–4 year age group, especially at age 3, may be a random phenomenon. This could become clearer after a longer observation period under the same conditions of ascertainment.

3. Temporary changes in host–environment interactions. Impressive demographic changes in relation to the health of children have occurred in The Netherlands during and before the period of study. Lower fertility rates (Table I) have resulted in a decline of the birth rate, smaller families and relatively more first-born children. In this period the proportion of immigrant children from (former) Dutch colonies as well as Mediterranean countries has risen from 5% to almost 15%. In general, housing conditions have further improved. The control of infectious diseases has improved through the various vaccination programmes, that for measles being the last to be introduced in 1976, and the use of more effective antibiotics. All this has undoubtedly led to a lower risk of infection and may thus have resulted in a lower mortality in the precancer phase (Kneale & Stewart, 1978).

Although the mortality rates for children in The Netherlands were already low in 1970, an impressive and steady decrease in these rates for the major causes of death, including infectious diseases, has since occurred (Table VII).

According to the results of an extensive population-based case-control study of (the same) children with ALL, diagnosed in the 1973–1979 period, a risk-increasing influence of 20% appeared to exist for higher socioeconomic classes and 100% for first-borns. A decreased risk of 30% was also established for exposure to serious infections in the first year of life (van Steensel-Moll *et al.*, 1986). Changes in host–environment interactions were therefore substantial and complex before and during the period of study. However, according to the data of this study their impact on the incidence of childhood leukaemia could only have been small.

4. Temporary changes in risk factors should be taken into account, especially for the birth cohort of 1978. From the case-control study of ALL-patients (van Steensel-Moll *et al.*, 1985*a*, *b*) relative risks of about 2 were indeed found for mothers exposed to prenatal X-rays, certain chemicals and pregnancy-saving hormones. However, the contribution of these factors to the risk of leukaemia could not exceed 10%. In the period 1973–1979, space–time clustering (van Steensel-Moll *et al.*, 1983*b*) proved to be virtually absent for young children with ALL. It can be concluded that there are no concrete clues in this respect, particularly not for the three small nuclear power plants.

**Table V** Distribution of immunological phenotype of childhood ALL in The Netherlands, according to WBC at diagnosis: 1979–1986

| *Number of leuocytes* × $10^9$ $l^{-1}$ (*WBC*) | *T-cell* | *Common ALL* | *Pre-B* | *B-cell* | *Unclassifiable*[a] | *No test result*[b] | *Total* | *No test done* |
|---|---|---|---|---|---|---|---|---|
| 0– 4.9 | 3 | 132 | 14 | 0 | 7 | 26 | 182 | 51 |
| 5–19.9 | 6 | 130 | 23 | 2 | 11 | 6 | 182 | 66 |
| 20–49.9 | 6 | 41 | 18 | 1 | 4 | 3 | 73 | 20 |
| 50–99.9 | 7 | 20 | 9 | 2 | 1 | 1 | 40 | 13 |
| >99.9 | 47 | 20 | 6 | 0 | 14 | 1 | 88 | 18 |
| Unknown | 1 | 2 | 0 | 0 | 0 | 0 | 3 | 2 |
| Total | 70 | 345 | 70 | 5 | 37 | 37 | 564 | 170 |
| Total (%) | 12.4 | 61.2 | 12.4 | 0.9 | 6.6 | 6.6 | 100 | – |

[a]Immunologically. [b]Due to lack of sufficient material.

**Table VI** Incidence of childhood leukaemia in North and Western Europe, the United States and Australia: From 1970

| | | Cases per $10^5$ per year | | | | |
|---|---|---|---|---|---|---|
| | | Per age group | | | | Ratio |
| *Population/country* | *Period* | *0–4* | *5–9* | *10–14* | *Adjusted 0–14* | *ALL ANLL* |
| USA/SEER whites | 73–82 | 6.8 | 3.5 | 2.3 | 4.4 | 5.4 |
| USA/SEER blacks | 73–82 | 3.4 | 2.0 | 2.0 | 2.5 | 2.9 |
| Australia | 77–82 | 7.4 | 3.6 | 2.2 | 4.6 | 5.3 |
| Finland | 71–82 | 5.4 | 3.0 | 2.7 | 3.9 | 5.0 |
| West Germany | 80–86 | 6.5 | 3.5 | 2.2 | 4.4 | 6.6 |
| Denmark | 80–84 | 5.7 | 2.7 | 2.8 | 3.9 | 5.3 |
| Manchester UK | 71–83 | 5.7 | 3.2 | 2.2 | 3.8 | 5.7 |
| LRF-survey[a] UK | 84–86 | 6.4 | 3.0 | 1.7 | 3.9 | 5.1 |
| The Netherlands | 73–86 | 6.1 | 3.1 | 1.9 | 3.9 | 8.3 |

[a]Cases from: S.W. Scotland, Cumbria, Yorkshire, Trent, S.W. England, S. Wales and E. Suffolk.

**Table VII** Relative change in childhood mortality in The Netherlands, 1970–1985: All causes of death (A) and infectious diseases (B)

| | Cases per $10^5$ per year by age group (1970–1972 = 100) | | | | | |
|---|---|---|---|---|---|---|
| | 0 years | | 1–4 years | | 5–9 years | |
| *Period* | *A* | *B* | *A* | *B* | *A* | *B* |
| 70–72 | 1215 | 79.4 | 81.1 | 11.5 | 41.8 | 2.1 |
| | % | % | % | % | % | % |
| 70–72 | 100 | 100 | 100 | 100 | 100 | 100 |
| 73–75 | 92 | 91 | 89 | 83 | 87 | 90 |
| 76–78 | 81 | 93 | 74 | 52 | 81 | 71 |
| 79–81 | 70 | 64 | 68 | 38 | 64 | 67 |
| 82–84 | 69 | 74 | 62 | 35 | 45 | 52 |
| 85–86 | 55 | 39 | 51 | 31 | 42 | 48 |

*Source:* CBS, Department of Health Statistics.

It can be concluded that there are no satisfactory explanations for either the observed temporary increase in the incidence of common-ALL in young children in The Netherlands or the difference in ALL in boys according to degree of urbanisation. In view of the suggested effects of demographic and socioeconomic changes on the incidence of lymphoproliferative diseases (Ramot *et al.*, 1984) and common aetiological and diagnostic aspects, the DCLSG has now started a study of the incidence of malignant lymphoma in children in The Netherlands in the same period.

We thank J.A. Bakkeren for supplying data on immunophenotyped cases in Nijmegen, Mrs M.Th. Verhagen-Teulings for supplying data from the Eindhoven cancer register, Margreet de Ruyter for coding, Leo Muller for his extensive work on the computer programs and G. Bieger-Smith for reviewing the English text.

### References

AUSTRALIAN PAEDIATRIC CANCER REGISTRY (1987). *Childhood Cancer in Australia: Incidence 1977–1982*. Department of Health, NSW: North Ryde.

BENNETT, J.M., CATOVSKY, D., DANIEL, M.TH. & 4 others (1976). Proposals for the classification of the acute leukaemias. *Br. J. Haematol.*, **33**, 451.

BIRCH, J.M., SWINDELL, R., MARSDEN, H.B. & MORRIS JONES, P.H. (1981). Childhood leukaemia in North West England 1954–77: Epidemiology, incidence and survival. *Br. J. Cancer.*, **43**, 324.

BRESLOW, N.E. & LANGHOLZ, B. (1983). Childhood cancer incidence: Geographical and temporal variations. *Int. J. Cancer*, **32**, 703.

FINNISH CANCER REGISTRY (1987). *Cancer Incidence in Finland, 1983*, p. 30. Cancer Society of Finland: Helsinki.

GREAVES, M., PEGRAM, S.M. & CHAN, L.C. (1985). Collaborative group study of epidemiology of acute lymphoblastic leukaemia subtypes: Background and first report. *Leuk. Res.*, **9**, 715.

GREAVES, M. (1986). Is spontaneous mutation the major 'cause' of childhood acute lymphoblastic leukaemia? *Br. J. Haematol.*, **64**, 1.

HAKULINEN, T., ANDERSEN, A.A., MALKER, B., PUKKALA, E., SCHOU, G. & TULINIUS, H. (1986). Trends in cancer incidence in the Nordic countries. *Acta. Pathol. Microbiol Scand.*[*A*], **94**, Suppl. 288.

KAATSCH, P. & MICHAELIS, J. (1987). *Jahresbericht 1986 über die kooperative Dokumentation von Malignomen in Kindesalter*. Institut für Medizinische Statistik und Dokumentation: Mainz.

KNEALE, G.W. & STEWART, A.M. (1978). Precancers and liability to other diseases. *Br. J. Cancer*, **37**, 448.

McKINNEY, P.A., CARTWRIGHT, R.A., SAIU, J.M.T. & 8 others (1987). The inter-regional epidemiological study of childhood cancer (IRESCC): A case control study of aetiological factors in leukaemia and lymphoma. *Arch. Dis. Child.*, **62**, 279.

PARKIN, D.M., STILLER, C.A., DRAPER, G.U. & 3 others (eds) (1988). *International Incidence of Childhood Cancer*, IARC Scientific Publications no. 87. IARC: Lyon.

RAMOT, B. & MAGRATH, I. (1982). The environment is a major determinant of the immunological sub-type of lymphoma and acute lymphoblastic leukaemia in children. *Br. J. Haematol.*, **52**, 183.

RAMOT, B., BASSOT, I.B., BRECHER, A. & ZAILOV, R. (1984). The epidemiology of childhood acute lymphoblastic leukaemia and non-Hodgkin lymphoma in Israel between 1976 and 1981. *Leuk. Res.*, **8**, 691.

SEER PROGRAM (1984). *Cancer Incidence and Mortality in the United States, 1973–1981*, NIH-publication 85-1837. National Cancer Institute: Bethesda, MD.

SIEGEL, S. (1956). *Nonparametric Statistics for the Behavioral Sciences*, pp. 52 and 252. McGraw-Hill: New York.

STILLER, C.A. (1985). Descriptive epidemiology of childhood leukaemia and lymphoma in Great Britain. *Leuk. Res.*, **9**, 671.

VAN DER REIJDEN, H.J., VAN WERING, E.R., VAN DE RIJN, J.M. & 4 others (1983). Immunological typing of ALL. *Scand. J. Haematol.*, **30**, 356.

VAN STEENSEL-MOLL, H.A., VALKENBURG, H.A. & VAN ZANEN, G.E. (1983*a*). Incidence of childhood leukaemia in the Netherlands. *Br. J. Cancer*, **47**, 471.

VAN STEENSEL-MOLL, H.A., VALKENBURG, H.A., VANDENBROUCKE, J.,P. & VAN ZANEN, G.E. (1983*b*). Time space distribution of childhood leukaemia in the Netherlands. *J. Epidemiol. Community Health*, **37**, 145.

VAN STEENSEL-MOLL, H.A., VALKENBURG, H.A. & VAN ZANEN, G.E. (1985*a*). Childhood leukaemia and parental occupation: A register-based case-control study. *Am. J. Epidemiol.*, **121**, 216.

VAN STEENSEL-MOLL, H.A., VALKENBURG, H.A., VANDENBROUCKE, J.P. & VAN ZANEN, G.E. (1985*b*). Are maternal fertility problems related to childhood leukaemia? *Int. J. Epidemiol.*, **14**, 555.

VAN STEENSEL-MOLL, H.A., VALKENBURG, H.A. & VAN ZANEN, G.E. (1986). Childhood leukaemia and infectious diseases in the first year of life: A register based case-control study. *Am. J. Epidemiol.*, **124**, 590.